# Persistent CMV IgM Without IgG Seroconversion in an Immunomodulated Pregnant Woman: A Case Report

**DOI:** 10.1155/crdi/1322365

**Published:** 2026-06-26

**Authors:** Francisco Vale, Rita Magano, Alice Alicerces, Inês Trabucho, Frederico Gonçalves, João Lourinho, Cláudia Silva Franco

**Affiliations:** ^1^ Infectious Diseases Department, Almada-Seixal Local Health Unit, Setúbal, Almada, Portugal; ^2^ Infectious Diseases University Clinic, Faculty of Medicine, University of Lisbon, Lisbon, Portugal, ulisboa.pt

**Keywords:** cytomegalovirus, immunomodulatory therapy, no seroconversion, pregnancy

## Abstract

**Background:**

Congenital cytomegalovirus (CMV) infection is the most common intrauterine infection worldwide. Maternal primary infection is diagnosed with presence of IgM antibodies, detection of IgG with low avidity index, and/or IgG seroconversion in sequential serum samples. We report the first case of a pregnant woman with persistent CMV IgM positivity without IgG seroconversion, which raises significant questions regarding immune response and fetal risk assessment.

**Case report:**

A 39‐year‐old woman, with a history of ankylosing spondylitis and biologic immunosuppression, exhibited persistent CMV IgM positivity and high‐level viremia without IgG seroconversion over two years of fertility support follow‐up. During her pregnancy, CMV IgM remained positive with transient viremia. Fetal evaluations were normal, and CMV DNA was not detected in amniotic fluid. The infant was born healthy, and, to date, no neurodevelopmental changes have been identified.

**Conclusion:**

This case underscores a rare immunological profile with significant implications for CMV diagnosis and fetal risk assessment. Persistent CMV IgM without IgG seroconversion, although rare, may occur in patients undergoing immunomodulatory therapies, complicating the interpretation of maternal and fetal risk. This scenario requires individualized care and a multidisciplinary approach.

## 1. Introduction

Human cytomegalovirus (CMV) infection is the leading cause of congenital viral infection globally, with a reported prevalence ranging from 0.2% to 2.2% [1]. Among neonates affected by this infection, approximately 15%–20% may develop long‐term neurodevelopmental sequelae, including sensorineural hearing loss [2].

Although universal preconception screening for CMV is not recommended in many international guidelines, baseline serology may be considered during preconception counseling in certain settings, since the absence of early testing often complicates interpretation of results during pregnancy [3]. In Portugal, however, the Directorate‐General of Health (DGS) recommends serological screening for CMV in all women attending preconception consultations [4].

The diagnosis of maternal primary infection (MPI) is generally established through the detection of IgG seroconversion observed in sequential serum samples and/or detection of CMV‐specific IgM associated with low IgG avidity [5].

CMV is a master of immune evasion, encoding numerous genes that subvert host defense mechanisms. It infects a broad range of cell types—including fibroblasts, epithelial and endothelial cells, monocytes, and T lymphocytes—and establishes lifelong latency with the capacity for periodic reactivation. Effective control of CMV relies on robust CD8^+^ T‐cell responses and humoral immunity: IgM appears early after infection, while IgG persists lifelong with progressively increasing avidity. Immunosuppressive therapies, particularly TNF‐α inhibitors, can impair class‐switch recombination (CSR) and germinal center formation, thereby reducing both IgG production and antibody avidity [6, 7].

In this report, we present an exceptional case characterized by persistent CMV IgM positivity without IgG seroconversion over a period of 2 years, including during pregnancy, despite confirmed prior viremia. This case raises significant challenges in immunological interpretation and in clinical impact.

## 2. Case Presentation

A 39‐year‐old woman with a diagnosis of ankylosing spondylitis (HLA‐B27 positive) received treatment with infliximab from 2015 to 2019, followed by adalimumab from 2019 onward. She underwent a fertility evaluation due to primary infertility.

Initial screening detected the presence of CMV IgM antibodies using the VIDAS CMV IgM assay (BioMérieux, France), while CMV IgG antibodies were absent as determined by the VIDAS CMV IgG assay, both performed using an enzyme‐linked fluorescent assay (ELFA).

Repeat testing 4 weeks later confirmed the persistence of CMV IgM antibodies by ELISA, along with the continued absence of CMV IgG antibodies, as assessed by ELFA at a different national laboratory.

This finding led to a delay in fertility procedures and prompted a referral to the Infectious Diseases department.

The patient reported a self‐limiting episode of hyperhidrosis 4 months prior, without any systemic or localized symptoms. Physical examination and laboratory evaluations, including complete blood count (CBC), metabolic panel, liver function tests, and thyroid function tests, were unremarkable, except for an elevated erythrocyte sedimentation rate (ESR) of 42 mm/hr. Antinuclear antibody (ANA) testing was positive at a titer of 1:640, exhibiting a homogeneous pattern.

Serological tests indicated past immunity to hepatitis A, hepatitis B, rubella, and Epstein–Barr virus (EBV). Herpes simplex virus Type 2 (HSV‐2) IgM was positive, while tests for *Toxoplasma gondii*, human immunodeficiency virus, hepatitis C virus, and syphilis were negative. A serum CMV real‐time polymerase chain reaction (PCR) test conducted in April 2022 revealed viremia with a viral load of 338,578 copies/mL.

Imaging studies and ophthalmologic evaluations excluded any organ involvement. Subsequent CMV PCR tests in May, July, and September 2022 were negative. At this stage, persistent CMV IgM positivity was observed, whereas CMV IgG remained undetectable when assessed using the Cobas 8000 system (Roche Diagnostics, Germany) based on a chemiluminescent immunoassay (CLIA).

In November 2023, the patient underwent embryo transfer, as more than 12 months had passed since the detection of CMV IgM without serological clearance, but with no evidence of active viremia. In the first trimester (January 2024), CMV IgM remained positive with detectable viremia (27,927 copies/mL) and an elevated ESR of 57 mm/hr, though no clinical symptoms were present. HSV‐2 IgM testing was negative. Follow‐up tests for CMV viremia in February and March were negative. Fetal ultrasound findings were normal. Amniocentesis at 24 weeks gestation showed no detectable CMV DNA.

The baby was delivered at term via caesarean section due to breech presentation, with a birth weight of 2850 g. Newborn CMV testing was not conducted, but an audiologic assessment performed in September 2024 yielded normal results. To date, no neurodevelopmental abnormalities have been identified.

## 3. Discussion

CMV infection during pregnancy poses a significant risk of vertical transmission, particularly in cases of MPI. Despite the lack of universal consensus for CMV screening during pregnancy, the European Congenital Cytomegalovirus Initiative (ECCI) has recommended early serological testing, with repeat testing every four weeks in seronegative women until 14–16 weeks’ gestation. This recommendation stems from recent evidence showing the efficacy of valacyclovir in preventing fetal infection when administered early after maternal infection [5, 8]. High‐avidity CMV IgG assays further enhance diagnostic precision, reliably excluding recent infections in the context of ambiguous IgM results [5].

In this case, we describe a unique clinical scenario involving a woman with persistent CMV IgM positivity, CMV viremia confirmed by PCR, and yet no seroconversion to CMV IgG over a period exceeding 2 years, including throughout pregnancy. To our knowledge, this represents the first reported case of confirmed CMV viremia with absent IgG seroconversion in the medical literature.

The persistence of IgM antibodies without IgG seroconversion is highly unusual. Typically, following acute infection, IgM levels decline within months, and IgG becomes detectable, persisting for life. However, CMV IgM assays are known for their low specificity, as IgM can remain elevated for extended periods or yield false‐positive results due to cross‐reactivity with other herpesviruses or interference from autoimmune antibodies [9, 10]. In our case, however, the presence of high CMV DNA viral loads confirmed active viral replication, ruling out a false‐positive IgM result and confirming a true CMV infection. Additionally, two serological techniques were performed to confirm the results obtained over time.

An important aspect to consider is the patient’s underlying autoimmune condition—ankylosing spondylitis—and her prolonged use of TNF‐α inhibitors, including infliximab and later adalimumab. These biologic agents are known to affect immune function, particularly B‐cell activity. Studies have shown that TNF‐α plays a crucial role in immunoglobulin CSR, a mechanism essential to produce IgG from IgM‐expressing B‐cells. Disruption of TNF‐α signaling through biologic therapy can impair CSR, potentially leading to insufficient IgG responses despite antigen exposure [11, 12].

Indeed, patients undergoing anti‐TNF‐α treatment have demonstrated blunted antibody responses to both natural infections and vaccinations, even when T‐cell responses remain intact [7, 13]. IgG avidity testing is essential in CMV diagnosis because antibody avidity increases through germinal‐center–driven affinity maturation, allowing discrimination between recent and past infection. Prior exposure to TNF‐α inhibitors may plausibly impair this process by disrupting germinal center B‐cell responses and IgG maturation. This is consistent with observations of attenuated antigen‐specific IgG and memory B‐cell responses during vaccination under TNF blockade, such as reported by Kummet et al., 2025 [7]. While our patient had normal IgG responses to rubella and hepatitis A and B, these antigens were likely encountered or immunized prior to the initiation of TNF‐blocker therapy. In contrast, CMV infection, which likely occurred after or during biologic treatment, may have faced an altered immunological environment that hindered effective B‐cell maturation and IgG production. Prior long‐term TNF‐α inhibitor therapy may have impaired germinal‐center–dependent B‐cell maturation, leading to delayed or absent CMV IgG seroconversion characterized by persistently low or intermediate avidity and quantitatively low IgG levels.

In terms of fetal risk, primary CMV infection in the first trimester is associated with a 23% risk of long‐term sequelae, while this risk drops substantially for second‐ and third‐trimester infections [14]. At the time of embryo transfer, the patient’s serological status was already under observation for nearly 2 years. Although the CMV viral load reappeared transiently in early pregnancy, its spontaneous clearance and the absence of IgG seroconversion suggested either a reactivation or reinfection, both of which are associated with significantly lower transmission risk compared to primary infection. Nonetheless, due to the rare immunological profile, we opted to perform amniocentesis to rule out fetal infection.

## 4. Conclusion

This case raises important clinical and immunological questions. Could TNF‐α blockade permanently impair humoral responses to specific antigens like CMV? What role might autoimmune conditions play in shaping these outcomes? And most importantly, how should we interpret CMV IgM positivity without IgG seroconversion in patients with long‐term biologic therapy? These questions underscore the need for individualized assessment of immune status and maternal‐fetal risk in similar clinical scenarios.

## Funding

This paper did not receive any funding.

## Consent

Patient consent was obtained.

## Conflicts of Interest

The authors declare no conflicts of interest.

## Data Availability

Data sharing is not applicable to this article as no datasets were generated or analyzed during the current study.
